# Supplementary material for: An improved differential evolution algorithm for multi-modal multi-objective optimization
Source: PeerJ Comput Sci. 2024 Mar 14;10:e1839. doi: 10.7717/peerj-cs.1839 (PMC11041989; doi:10.7717/peerj-cs.1839)
Supplement: Supplemental Information 2 [file peerj-cs-10-1839-s002.pdf]

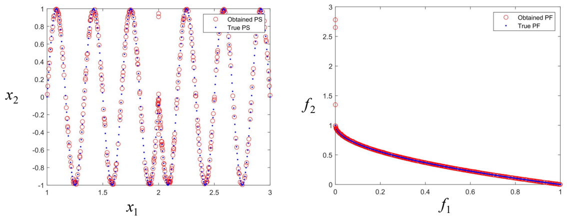

MMF1

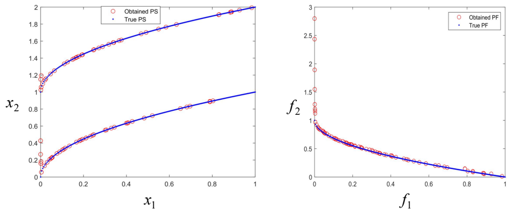

MMF2

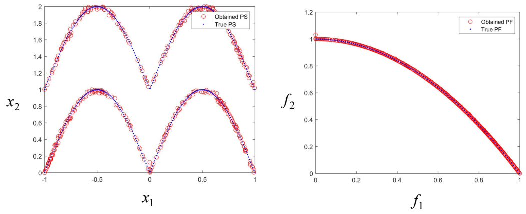

MMF4

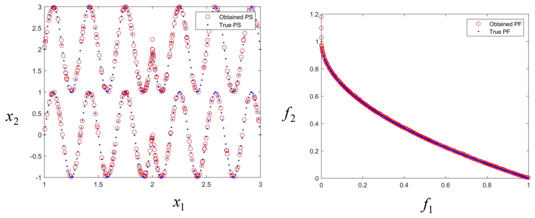

MMF5

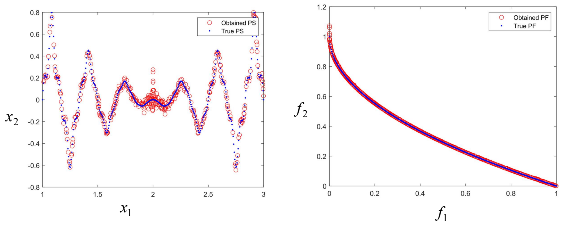

MMF7

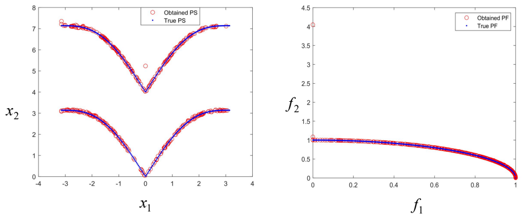

MMF8

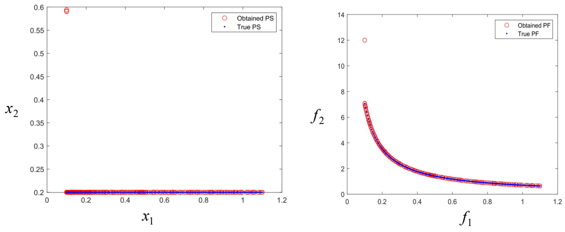

MMF10

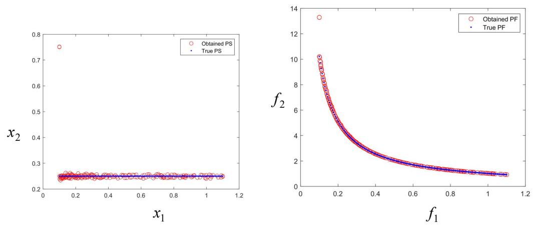

MMF11

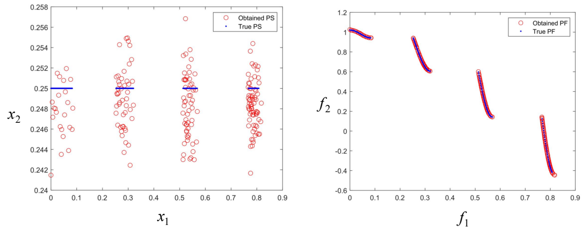

MMF12

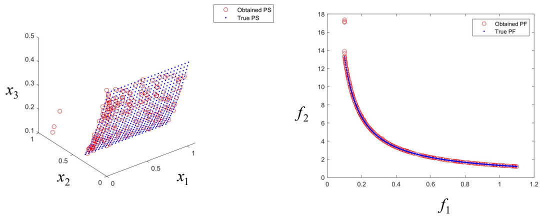

MMF13

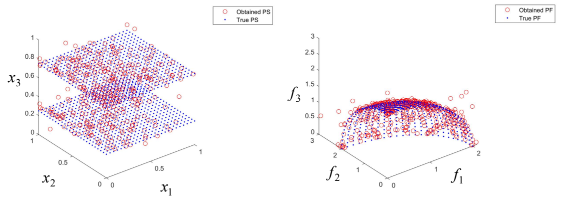

MMF14

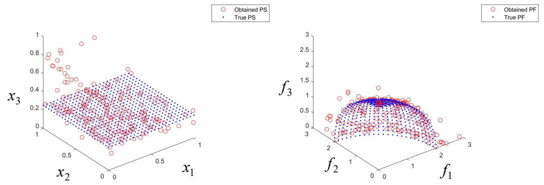

MMF15

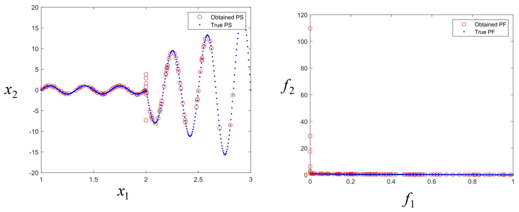

MMF1_e

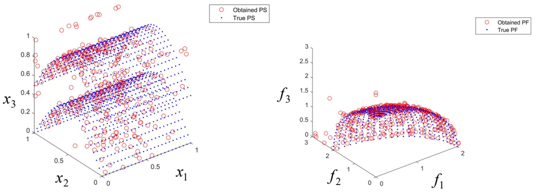

MMF14_a

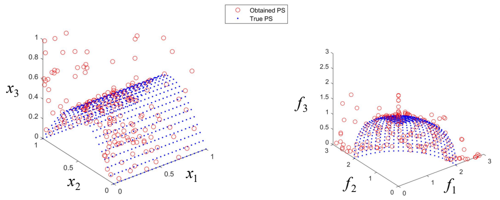

MMF15_a

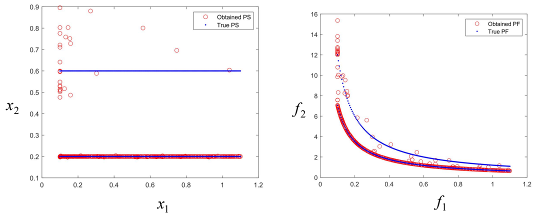

MMF10_l

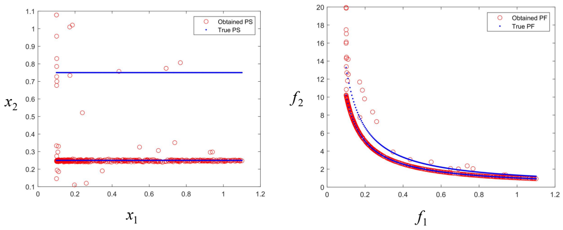

MMF11_l

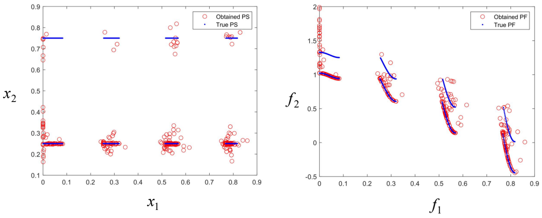

MMF12_l

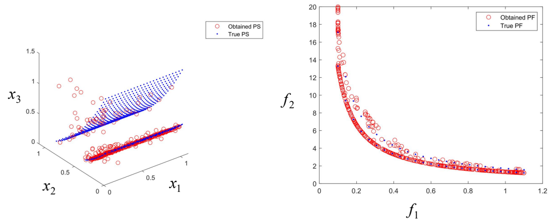

MMF13_l

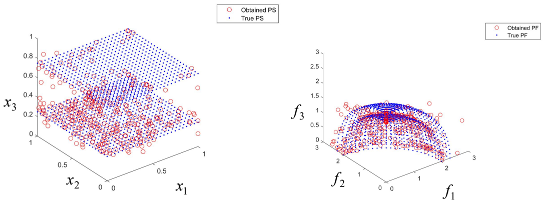

MMF15_l

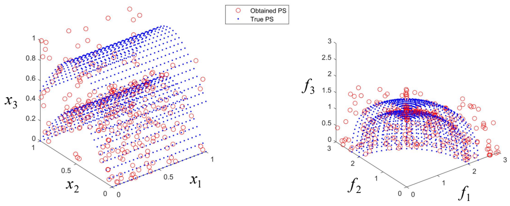

MMF15_a_l

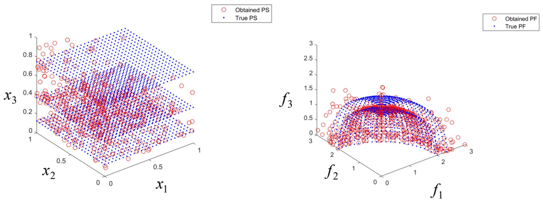

MMF16_l1

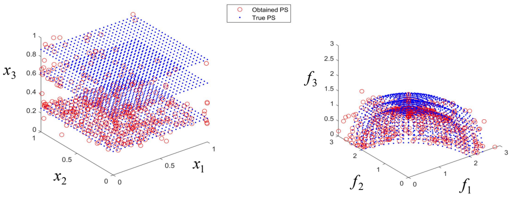

MMF16_l2

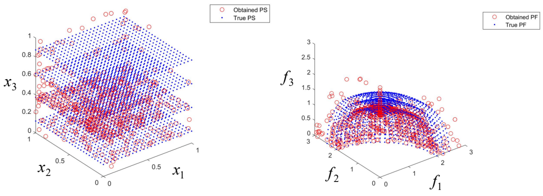

MMF16_l3
