# Supplementary figures and images for: An improved differential evolution algorithm for multi-modal multi-objective optimization
Source: PeerJ Comput Sci. 2024 Mar 14;10:e1839. doi: 10.7717/peerj-cs.1839 (PMC11041989; doi:10.7717/peerj-cs.1839)

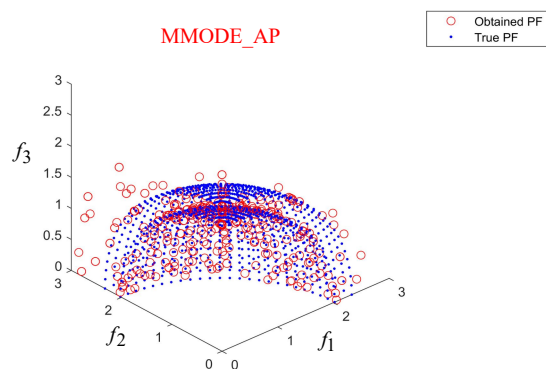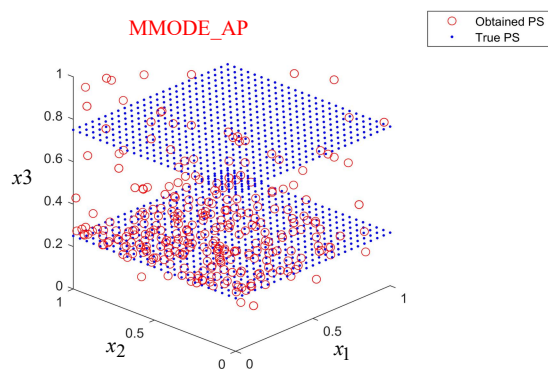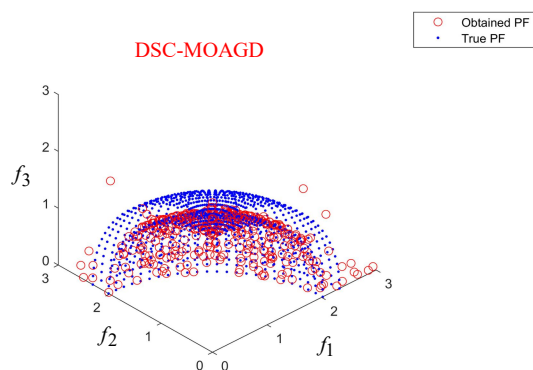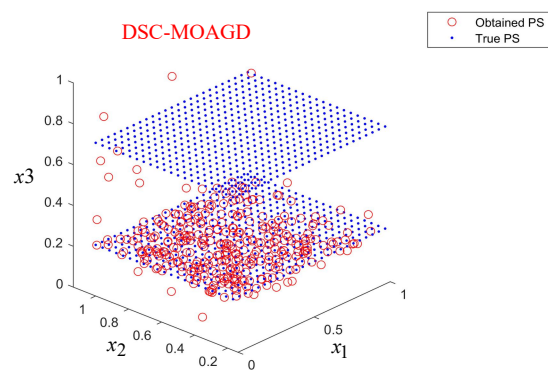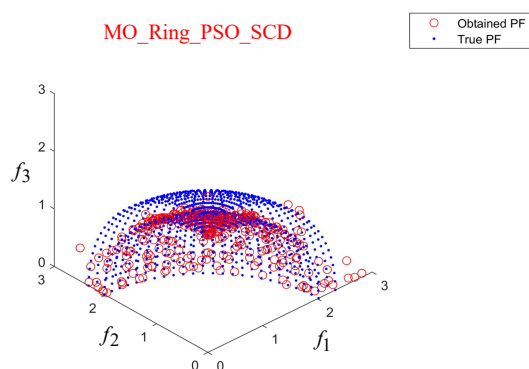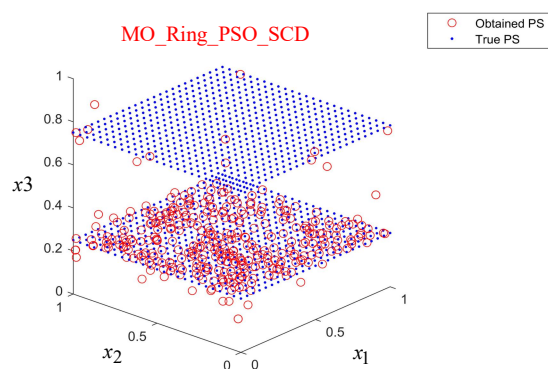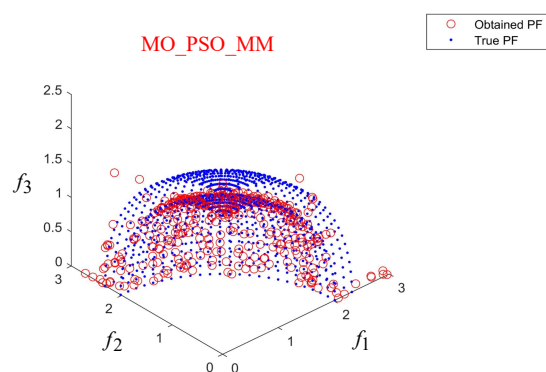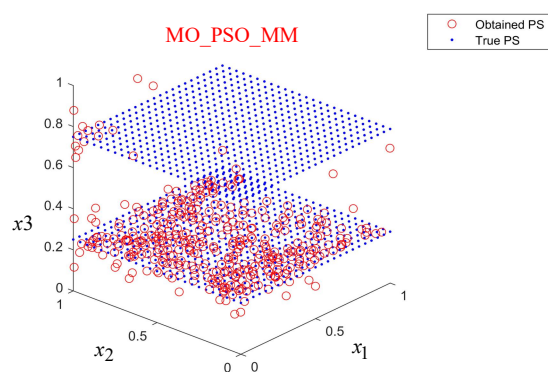

Supplement: Supplemental Information 3 [file peerj-cs-10-1839-s003.pdf]

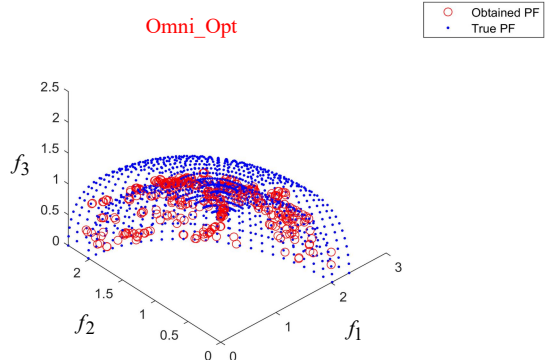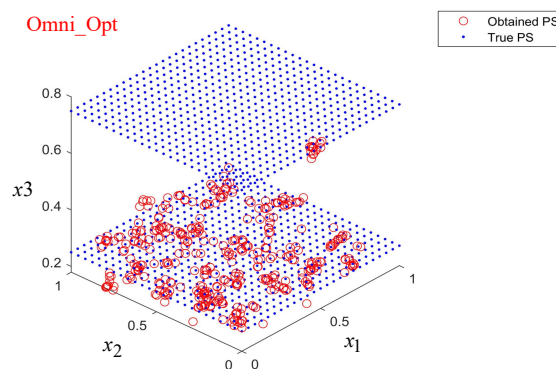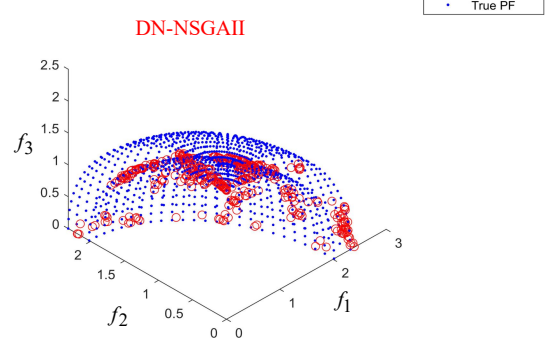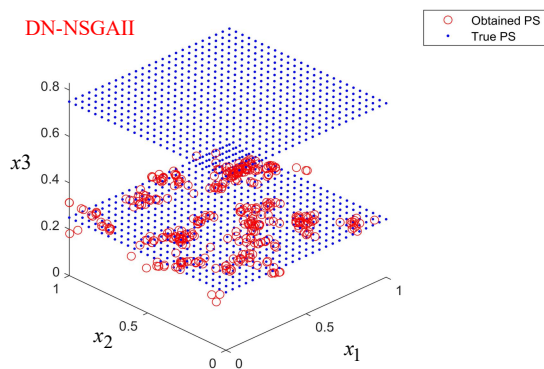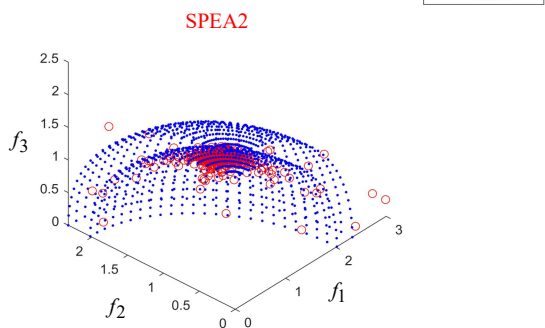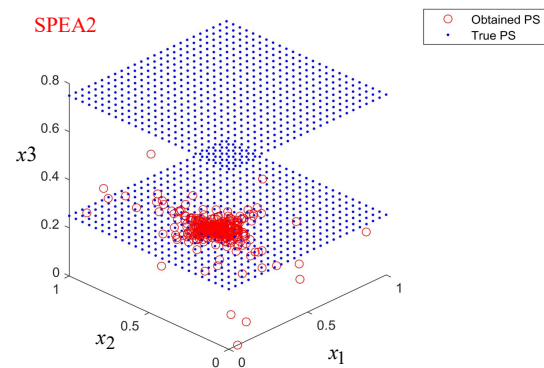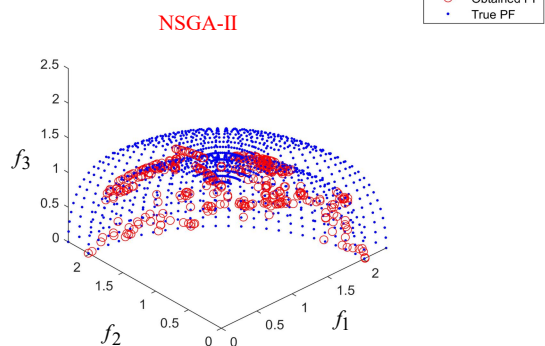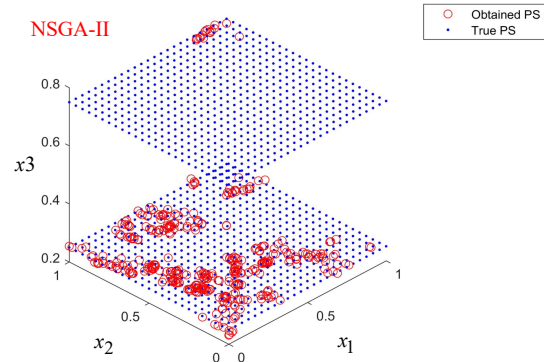

Supplement: Supplemental Information 4 [file peerj-cs-10-1839-s004.pdf]
