# Supplementary material for: An improved differential evolution algorithm for multi-modal multi-objective optimization
Source: PeerJ Comput Sci. 2024 Mar 14;10:e1839. doi: 10.7717/peerj-cs.1839 (PMC11041989; doi:10.7717/peerj-cs.1839)
Supplement: Supplemental Information 5 [file peerj-cs-10-1839-s005.docx]

| Datasets | URL |
| --- | --- |
| CEC'2020 benchmark functions. | https://github.com/P-N-Suganthan/2020-Multimodal-Multi-Objective-Benchmark |
